# 

**Published:** 1851-07

**Authors:** 


### NOTICE TO SUBSCRIBERS.

Those of our subsubscribers who have paid Mr. Wood, our agent, may understand that want of room on the cover, is the reason that their receipts are not acknowledged in this number.

We would say to our subscribers who have not yet paid, that we have a few numbers of the Intelligencer for June left, which will be forwarded to those who pay soon. After they are gone, any subscriber can have the numbers to be issued after the time of payment by forwarding his subscription to the Journal—but no back numbers.

Our list of receipts ought to be as long each number of the Journal as it is this month, if our subscribers would pay up what they owe us. We are, however, very thankful to those who do pay.

### RECEIPTS FOR THE JOURNAL FOR MAY AND JUNE.

| | |
|---|---|
| **ILLINOIS.** | |
| Drs. James Woodward, | $5 00 |
| W. B. Norton, | 2 00 |
| Davis & Balch, | 2 00 |
| E. G. Mygatt, | 4 00 |
| D. Ray, | 2 00 |
| C. W. Knott, | 2 00 |
| R. F. Henry, | 2 00 |
| George S. Wheeler, | 2 00 |
| E. D. Whiting, | 3 00 |
| W. P. Richardson, | 4 00 |
| Joseph Tefft, | 4 00 |
| H. Rosenkrans, | 2 00 |
| J. P. Walker, | 3 00 |
| E. E. Webster, | 2 00 |
| Ezekiel Friend, | 2 00 |
| Ezekiel Keenon, | 2 00 |
| D. D. Thompson, | 2 00 |
| J. Pearson, | 2 35 |
| A. Hull; | 2 00 |
| A. Berchlemann, | 2 00 |
| J. Randolph, | 4 00 |
| Samuel Thompson, | 2 00 |
| Van Petten & French, | 2 00 |
| J. M. Logan, | 4 00 |
| S. R. Mason, | 2 00 |
| B. S. Barrows, | 3 00 |
| H. A. Parkhurst, | 2 00 |
| J. H. Harrod, | 4 00 |
| J. V. Goltra, | 2 00 |
| John Warner, | 2 00 |
| R. Morris, | 2 00 |
| W. W. Sedgwick, | 2 00 |
| J. J. Beatty, | 2 00 |
| H. Hitchcock, | 2 00 |
| James Smick, | 2 00 |
| A. J. Gilbert, | 2 50 |
| N. S. Anderson, | 2 00 |
| E. Smith, | 2 00 |
| U. P. Golliday, | 2 00 |
| A. Hull, | 2 00 |
| P. C. Mosier, | 2 00 |
| L. Nutting, | 2 00 |
| G. W. P. Harding, | 2 00 |
| J. Arnold, | 4 00 |
| J. W. Spalding, | 2 00 |
| C. N. Andrews, | 4 00 |
| W. C. Snyder, | 2 00 |
| John Walker, | 2 00 |
| Thomas Stanton, | 2 00 |
| David Newell, | 2 00 |
| Sawyer, | 4 00 |
| L. D. Boone, | 4 00 |
| J. C. Patterson, | 2 00 |
| L. Q. Newton, | 4 00 |
| Messrs. Clarke & Co., | 3 00 |
| Drs. Townsend Seely, | 4 00 |
| J. Brinckerhoff, | 2 00 |
| R. T. Goodwin, | 2 00 |
| E. A. Rogers, | 2 00 |
| J. Ogden, | 2 00 |
| Charles Gorham, | 3 00 |
| Drs. Charles H. Harrison, | $2 00 |
| Ira C. Bardwell, | 2 00 |
| A. M. Catlin, | 8 00 |
| Wood & Wilson, | 2 00 |
| —— Rogers, | 6 00 |
| H. P. Kelso, | 2 50 |
| P. S. Secor, | 2 00 |
| J. H. Piersol, | 2 00 |
| J. Gregory, | 2 00 |
| *W.* C. Quigley, | 2 33 |
| T. L. Fitch, | 4 00 |
| H. G. Ager, | 2 00 |
| Thomas L. Barnes, | 2 00 |
| H. P. Griswold, | 2 00 |
| C. Goodbrake, | 2 00 |
| J. B. E. Albright, | 2 00 |
| W. M. Sweetland, | 4 00 |
| B. F. Stephenson, | 2 00 |
| J. P. Dimmitt, | 2 00 |
| **WISCONSIN.** | |
| Drs. A. & E. N. Clarke, | $2 00 |
| J. R. Bradway, | 2 00 |
| James S. Russell, | 2 00 |
| J. H. Turner, | 2 00 |
| A. H. Van Orstrandt, | 2 00 |
| John R. Goodno, | 4 00 |
| C. C. Warner, | 2 00 |
| Russell Parkhurst, | 5 00 |
| W. Law, | 2 00 |
| H. B. Willard, | 2 00 |
| Robert W. Earll, | 2 00 |
| H. W. Hunt, | 4 00 |
| N. C. Rowley, | 2 00 |
| S. W. Abbott, | 2 00 |
| W. H. Walker, | 4 00 |
| D. Robinson, | 5 00 |
| J. W. Brown, | 2 00 |
| F. Paddock, | 5 00 |
| G. W. Lee, | 2 00 |
| J. H. Morrison, | 4 00 |
| Hays McKinley, | 2 00 |
| Clarke & Rice, | 2 00 |
| L. E. Hackley, | 2 00 |
| R. T. Hayes, | 2 00 |
| John Bartlett, | 6 00 |
| W. R. Fowler, | 2 00 |
| J. B. Selby, | 6 00 |
| S. H. Graves, | 8 00 |
| D. Walker, | 2 00 |
| J. Parker, | 2 00 |
| F. Paddock, | 4 00 |
| S. Blood, | 4 00 |
| J. B. Fleming, | 2 00 |
| T. Treat, | 2 00 |
| E. Rider. | 4 00 |
| L. J. Barrows, | 4 00 |
| C. Bicknell, | 5 00 |
| George Riddell, | 1 00 |
| R. C. Rockwood, | 2 00 |
| W. R. Streeter, | 1 00 |
| E. B. West, | 6 00 |
| H. W. Johnston, | 4 00 |

### RECEIPTS FOR MAY AND JUNE CONTINUED.

WISCONSIN CONTINUED.

| | |
|---|---|
| Drs. L. Nash, | $3 00 |
| L. Dennison | 2 00 |
| G. Wright, | 3 00 |
| O. B. Pearson, | 1 00 |
| Charles Cowles, | 2 00 |
| C. Ware, | 2 00 |
| D. H. Shumway, | 1 00 |
| J. C. Cole, | 1 00 |
| A. H. Bowe, | 3 00 |
| J. Look, | 2 00 |
| W. Torrey, | 4 00 |
| B. Noyes, | 6 00 |
| R. Hull, | 4 00 |
| S. Secor, | 1 00 |
| Wm. Aainsworth, | 1 00 |
| C. Nettleton, | 2 00 |
| C. D. Vilas, | 2 00 |
| A. P. Adams, | 2 00 |
| L. B. W. Johnson, | 2 00 |

INDIANA.

| | |
|---|---|
| Drs. James A. Wood, | 2 00 |
| J. P. Cobbs, | 2 00 |
| Charles Badger, | 2 00 |
| J B. Buchtel, | 2 00 |
| W. C. Matchett, | 6 00 |
| Wm. Wickham, | 4 00 |
| S. B. Kiler, | 4 00 |
| L. H. Sovereign, | 2 00 |
| Wm. Clifford, | 2 00 |
| E. Clifford, | 4 00 |
| A. Buchanan, | 4 00 |
| U. B. Tingley, | 2 00 |
| S. W. Purviance, | 2 00 |
| H. T. Snook, | 2 00 |
| M. W. Thomas, | 2 00 |
| J. P. Tucker, | 2 00 |
| H. Wehmer, | 5 00 |
| A. L. Hamer, | 2 00 |
| J. E. Heald, | 4 00 |
| J. H. Paxton, | 2 00 |
| A. D. Sarjent, | 5 00 |
| T. Bullard, | 2 00 |
| S. W. Ritchey, | 2 00 |
| J. H. Buell, | 4 00 |
| J. O. Wade, | 4 00 |
| R. M. Gilkison, | 2 00 |
| A. J. Mirick, | 4 00 |
| Samuel Reifenberick, | 2 00 |
| J. Pierce, Esq., | 1 00 |
| Drs. J. B. Hedges, | 2 00 |
| H. G. & M. Sexton, | 6 00 |
| J. DePew, | 2 00 |
| J. W. McKinney, | 1 00 |
| O. H. Martin, | 2 00 |
| Jefferson Miller, | 2 00 |
| George Manners, | 2 00 |
| H. Bussey, | 2 00 |
| Thomas W. Mellen, | 2 00 |

MICHIGAN.

| | |
|---|---|
| Drs. Thomas Brayton, | $2 00 |
| J. Loomis, | 2 00 |
| Drs. B. Hudson, | $2 00 |
| Wm. Romine, | 5 00 |
| J. A. Mason, | 2 00 |
| Matthew Gill, | 2 00 |
| Campbell & Cox, | 2 00 |
| Lafayette Lovell, | 2 00 |
| Ezra Stetson, | 6 00 |
| A. W. Mack, | 2 00 |
| B. Hard, | 88 |
| E. H. Keables, | 2 40 |
| Jacob Allen, | 7 00 |
| J. K. Finley, | 2 00 |
| Jos. Mansfield, | 5 00 |
| S. Richardson, | 6 00 |
| S. Stodard, | 4 00 |
| H. Lockwood, | 3 00 |
| Edwin Stewart, | 2 00 |
| S. D. Richardson, | 2 00 |
| Charles W. Brown, | 2 00 |
| Hiram Sheldon, | 2 00 |
| Andrew Foster, | 2 00 |
| John W. Hall, | 2 00 |
| L. B. Coates, | 2 00 |
| Wm. Upjohn, | 2 00 |
| C. B. Goodrich, | 2 00 |
| Daniel Peck, | 2 00 |
| R. S. Hawley, | 2 00 |
| A. Murray, | 2 00 |
| J. N. Chase, | 2 00 |
| Tompkins & Garwood, | 1 00 |
| D. E. Brown 2 00. Z. T. Slater | 2 00 |

IOWA.

| | |
|---|---|
| Drs. E. R. Ford, | 2 00 |
| —— Rivereau, | 2 00 |
| J. M. Anderson, | 4 00 |
| N, Steel, | 2 00 |
| J. Q. H. Scott, | 2 00 |
| Daniel Dickinson, | 4 00 |
| Asa Horr, | 2 00 |
| A. A. Hemenway, | 3 00 |
| E. M. Downs, | 5 00 |
| J. Doron, | 2 00 |
| W. H. Rousseau, | 4 00 |
| J. M. Weatherwax, | 2 00 |
| J, D. Blanchard, | 2 00 |
| Robert McGannin, | 2 00 |
| F. Brady, | 1 00 |

OHIO.

| | |
|---|---|
| Drs. P. Beaugrand, | $2 00 |
| A. W. Dewey, | 5 00 |
| Lathrop & Woodward, | 4 00 |
| A. R. Ferguson, | 2 00 |
| H. Burritt, | 2 00 |
| Nathaniel Strong, | 4 00 |
| S. Stough, | 2 00 |
| W. W. Stilson, | 2 00 |
| S. Fisher, | 2 00 |

NEW YORK.

| | |
|---|---|
| Albany Medical College, | 2 00 |
| Dr, R. B. Landon, | 2 00 |

MINNESOTA.

| | |
|---|---|
| Dr. V. Fell, | $5 00 |

